# The Genome of the Marine Alga *Ulva compressa* (Chlorophyta) Reveals Protein-Coding Genes with Similarity to Plants and Green Microalgae, but Also to Animal, Bacterial, and Fungal Genes

**DOI:** 10.3390/ijms23137279

**Published:** 2022-06-30

**Authors:** Héctor Osorio, Patricio Tapia-Reyes, Daniela Espinoza, Daniel Laporte, Alberto González, Eduardo Castro-Nallar, Alejandra Moenne

**Affiliations:** 1 Marine Biotechnology Laboratory, Faculty of Chemistry and Biology, University of Santiago of Chile, Alameda 3363, Santiago 9170022, Chile; hector.osorio@usach.cl (H.O.); daniela.espinoza@usach.cl (D.E.); alberto.gonzalezfi@usach.cl (A.G.); 2 Center of Bioinformatics and Integrative Biology, Faculty of Life Sciences, University Andres Bello, República 330, Santiago 8370186, Chile; patricio.tapia.bioinfo@gmail.com; 3 Laboratorio Multidisciplinario, Instituto de Ciencias Biomédicas, Universidad Autónoma de Chile, Talca 3467987, Chile; daniel.laporte@uautonoma.cl; 4 Departamento de Microbiología, Facultad de Ciencias de la Salud, Universidad de Talca, Campus Talca, Av. Lircay s/n, Talca 3460000, Chile; ecastron@utalca.cl; 5 Centro de Ecología Integrativa, Universidad de Talca, Campus Talca, Av. Lircay s/n, Talca 3460000, Chile

**Keywords:** genome, protein-coding genes, structural RNA genes, marine alga, *Ulva compressa*

## Abstract

The genome of the marine alga *Ulva compressa* was assembled using long and short reads. The genome assembly was 80.8 Mb in size and encoded 19,207 protein-coding genes. Several genes encoding antioxidant enzymes and a few genes encoding enzymes that synthesize ascorbate and glutathione were identified, showing similarity to plant and bacterial enzymes. Additionally, several genes encoding signal transduction protein kinases, such as MAPKs, CDPKS, CBLPKs, and CaMKs, were also detected, showing similarity to plants, green microalgae, and bacterial proteins. Regulatory transcription factors, such as ethylene- and ABA-responsive factors, MYB, WRKY, and HSTF, were also present and showed similarity to plant and green microalgae transcription factors. Genes encoding enzymes that synthesize ACC and ABA-aldehyde were also identified, but oxidases that synthesize ethylene and ABA, as well as enzymes that synthesize other plant hormones, were absent. Interestingly, genes involved in plant cell wall synthesis and proteins related to animal extracellular matrix were also detected. Genes encoding cyclins and CDKs were also found, and CDKs showed similarity to animal and fungal CDKs. Few genes encoding voltage-dependent calcium channels and ionotropic glutamate receptors were identified as showing similarity to animal channels. Genes encoding Transient Receptor Potential (TRP) channels were not identified, even though TRPs have been experimentally detected, indicating that the genome is not yet complete. Thus, protein-coding genes present in the genome of *U. compressa* showed similarity to plant and green microalgae, but also to animal, bacterial, and fungal genes.

## 1. Introduction

Marine algae can be classified as green, red, and brown algae, where green and red algae are more closely related to each other since they belong to Plantae, while brown algae belong to Stramenopiles [1]. The first genome sequenced from a marine alga was the genome of the brown alga *Ectocarpus siliculosus*, which showed a size of 214 Mb and 16,256 protein-coding genes [2]. Other genomes of brown macroalgae have been described, such as those of *Saccharina japonica*, having a size of 537 Mb and 18,733 protein-coding genes [3] and *Undaria pinnatifida*, having a size of 539 Mb and 14,178 protein-coding genes [4]. Thus, the genomes of brown macroalgae sequenced to date vary in size from 214–539 Mb and they encode 14,000 to 18,000 protein-coding genes. The first genome sequenced from a red macroalga was the genome of *Chondrus crispus*, showing a size of 105 Mb and 9606 protein-coding genes [5]. Other genomes of red macroalgae have been described, such as those of the *Pyropia yezoensis* genome (43 Mb and 10,327 protein-coding genes) [6], *Porphyra umbilicalis* (87.7 Mb and 13,125 protein-coding genes) [7], *Gracilariopsis chorda* (92.1 Mb and 10,806 protein-coding genes) [8], *Gracilariopsis lemaneiformis* (38.73 Mb and 9281 protein-coding genes) [9], and *Pyropia haitanensis* (53.3 Mb and 10,903 protein-coding genes [10]. Thus, the genomes of red macroalgae sequenced to date show a size of 30–100 Mb and encode 9000–13,000 protein-coding genes. The first sequenced green macroalga was of the genome *Ulva mutabilis*, and it showed a size of 98.5 Mb and 12,924 protein-coding genes [11]. Then, the genome *Caulerpa lentilifera* was sequenced and showed a size of 26 Mb and 9311 protein-coding genes [12]. Thus, green macroalgae genomes sequenced to date have a size ranging from 26–98.5 Mpb and encode 9000–13,000 protein-coding genes. Therefore, genome size and number of protein-coding genes are more similar in green and red algae, and genome size and protein gene number are higher in brown algae.

Green algae (Cholorophyta) can be divided in Ulvophyceae, Chlorophyceae, Prasinophyceae, Chlorodendrophyceae, Trebouxophyceae, and Charophyceae. Ulvophyceae are marine macroalgae, such as *U. compressa*, *U. lactuca*, *U. mutabilis*, *U. fasciata*, and others [13]. The marine macroalga *U. compressa* has been extensively studied over the last 20 years due to its ability to accumulate heavy metals such as copper, zinc, iron, cadmium [14,15,16], and nickel (A. González, unpublished). It has been shown that *U. compressa*, cultivated in vitro with increasing concentrations of copper, such as 2.5, 5, 7.5 and 10 µM for 0 to 12 d, displays an increase in the level of glutathione (GSH) and phytochelatins (PCs), which are peptides of condensed GSH (*n* = 2–4), and in the expression of metallothioneins (MTs), which are small, cysteine-rich proteins that bind heavy metals [17]. Recently, it was shown that copper accumulates in the chloroplasts of *U. compressa* as electrodense nanoparticles containing copper and sulfur, suggesting that copper is probably bound to sulfide, GSH, PCs, and/or MTs [18]. In addition, three MTs from *U. compressa*, named UcMT1, UcMT2, and UcMT3, have been cloned and expressed in bacteria allowing for an increased accumulation of copper and zinc [19], suggesting that these proteins may participate in the accumulation of copper in *U. compressa*.

*U. compressa* cultivated with copper and cadmium exhibit an oxidative stress condition, characterized by the accumulation of superoxide anions and hydrogen peroxide [20,21]. To mitigate the oxidative stress condition, the expression and activity of antioxidant enzymes, such as superoxide dismutase (SOD), ascorbate peroxidase (AP), glutathione reductase (GR), and peroxiredoxin (PRX), were increased in [20,21]. The main antioxidant enzyme is AP since its activity exponentially increases in response to copper stress from day 3 to day 7 of culture [14]. Moreover, a strong AP activity, and no catalase (CAT) activity, has been detected in *U. compressa* from copper-polluted sites [14]. Furthermore, copper induces increased activity in enzymes involved in ascorbate (ASC) synthesis, such as L-gulonolactone dehydrogenase (L-GLDH) and L-galactose dehydrogenase (L-GDH), and GSH synthesis, such as glutamate-cysteine synthase (GCS) and glutathione synthase (GS) [22]. In addition, basal metabolism enzymes, such as pyruvate dehydrogenase (PDH), isocitrate dehydrogenase (IDH), and 2-oxoglutarate dehydrogenase (OGDH), have also been shown to increase in response to copper excess [21], as well as the enzyme of secondary metabolism, phenylalanine ammonia lyase (PAL) [20].

In *U. compressa*, the activation of the expression of antioxidant enzymes requires the activation of signal transduction protein kinases (PKs) such mitogen-activated PKs (MAPKs), which are activated by Reactive Oxygen Species [23] and Calcium-Dependent PKs (CDPKs), calcineurin B-like PKs (CBLPKs) and calcium/calmodulin PKs (CaMPK), which are activated by intracellular calcium release [24,25,26]. Intracellular calcium release is due to initial extracellular calcium entry through transient receptor potential (TRP) channels and voltage-dependent calcium channels (VDCCs) located in the cellular membrane of *U. compressa* [27,28]. The activation of TRPs provides entry to extracellular calcium and copper ions, leading to depolarization events that activate VDCCs, allowing additional extracellular calcium entry [28]. In addition, the increase in intracellular calcium activates the calcium/ryanodine-dependent channel and the release of IP3 from cellular membrane activate inositol triphosphate (IP3)-dependent channels. This leads to the release of calcium from the endoplasmic reticulum (ER) to the cytoplasm [25]. The activation of VDCCs also involves the activation of cAMP-dependent PKs (PKAs) and cGMP-dependent PKs (PKGs) [28]. Moreover, the initial activation of TRPs also induces the release of amino acids, serotonin, and dopamine, which activate glutamate-, serotonin-, and dopamine-like receptors. These, in turn, activate other TRP channels that finally activate VDCCs, reinforcing extracellular calcium entry in *U. compressa* [29]. To identify the key genes that encode the proteins and enzymes involved in copper tolerance and accumulation, it is important to sequence the genome of *U. compressa* as well as promoter sequences of protein-coding genes. In the future, these genes could be transferred to terrestrial plants to remediate heavy metals in contaminated soils located near copper mines or copper smelters.

In this work, we sequenced the genome of *U. compressa* using PacBio and Illumina techniques, assembled the genome, and annotated protein-coding genes in order to identify which genes encode antioxidant enzymes, enzymes that synthesize ASC and GSH, MTs, signal transduction protein kinases, transcription factors, enzymes involved in phytohormone synthesis, proteins that regulate cell cycle, proteins for cell wall synthesis, and proteins involved in extracellular calcium entry and intracellular calcium release.

## 2. Results and Discussion

### 2.1. U. compressa Genome Assembly, Annotation of Protein-Coding Genes and Comparison with Other Green Algae Genomes

The genome of *U. compressa* was sequenced, and 5.3 Gb of PacBio sequences (976,831 contigs) and 10.7 Gb of Illumina sequences (68,858,316 reads) were obtained. Contigs of PacBio and reads of Illumina were assembled in 2601 scaffolds using the MaSuRCA software, and a genome of 80.8 Mb was obtained showing a GC content of 57.3% (Table 1). The total size of the genome was estimated to be 122 Mb and the heterozygosity was found to be 1.64% using the GENOMESCOPE software. The protein-coding genes were predicted using the MAKER software, and they were 19,207. Using the STAR software, we found that 17,173 genes (89%) could be confirmed using transcriptomic data, previously obtained from *U. compressa* [24,30,31], and 16,118 genes (84%) aligned with at least five reads of these transcriptomes, thus validating most predicted protein-coding genes. The core protein-coding genes were identified using the BUSCO software, and there were 1519 core genes, of which 1207 (80%) were complete genes, 1183 (79%) were single copy and complete genes, 24 were complete and duplicated genes, 40 were fragmented genes, and 272 were missing. The protein-coding genes showed an average length of 3392 bp, the exons showed an average length of 322 bp, the introns showed an average length of 479 nucleotides, and the number of introns per gene was 3.6. The 3′UTR regions showed an average length of 333 bp, while the 5’UTR regions displayed an average length of 557 bp. In addition, of the 19,207 protein-coding genes, only 7523 genes showed similarity to known protein-coding genes present in the NCBI NR database, representing 39.2% of total protein-coding genes, indicating that 60.8% of total genes are unknown.

Novel repeated sequences were identified using MAKER, and known repeated sequences were detected using the REPEATMASKER software and REPBASE database; they represented 18.9% of the genome. Repeated sequences corresponded to LINEs (1.77%), LTRs (1.52%), and DNA elements (0.61%) where 15.04% of the repeated sequences were unknown. Structural RNAs were identified using the StructRNAFinder software, and they corresponded to 18 rRNAs, including 28S, 18S, 5.8S, and 5S; 109 tRNAs; 27 snoRNAs involved in the methylation of rRNAs; 11 snRNAs involved in the splicing of mRNAs; and 87 primary miRNAs belonging to 49 families that are involved in the post-transcriptional regulation of gene expression (Table 1). In this sense, the *U. compressa* genome encodes four Dicer proteins and one Argonaute protein (data not shown).

Regarding genome size and protein-coding gene number, the genome of *U. mutabilis* showed a size of 98.5 Mb (57.2% of GC) and contained 12,924 protein-coding genes. Genes supported by Illumina transcriptome sequences represented 91.8%, and the percentage of core genes found with a BUSCO was 92% [11] (Table 1). In addition, repeated sequences represented 35% of the *U. mutabilis* genome, whereas in the *U. compressa* genome, they were only 18.9% (Table 1). Thus, the *U. mutabilis* genome showed a higher size than the *U. compressa* genome but a lower number of protein-coding genes. In addition, the genome of the green microalga *Chlamydomonas reinhardtii* showed a size of 121 Mb and 15,143 protein-coding genes [32]. Thus, the unicellular *C. reinhartii* showed a higher genome size and a higher number of genes than the multicellular alga *U. mutabilis*, indicating that genome size and the number of protein-coding genes do not explain the transition to multicellularity in green algae. In this sense, it has been recently reported that the transit of algae to multicellularity and then to land was not only due to an increased number of genes that have been gained, but also to an increased number of genes that have been lost [33].

### 2.2. Classification of Protein-Coding Genes in U. compressa Genome

**Figure 1:**
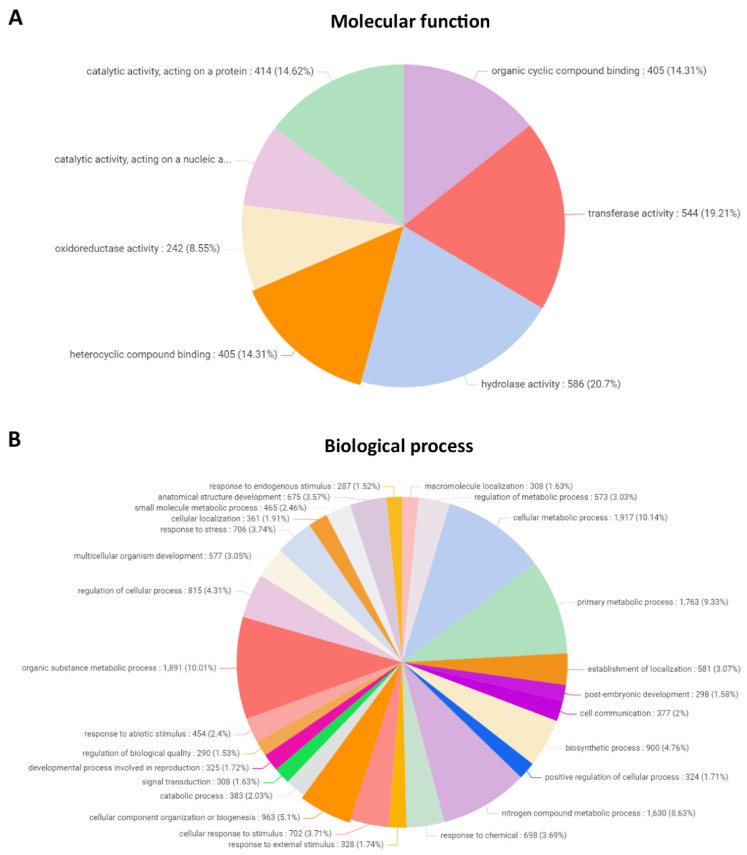
Classification into molecular function (**A**) and biological process (gene ontology) (**B**) of protein-coding genes in the genome of *U. compressa*.

The 7523 proteins having similarity to known protein-encoding genes were classified considering their molecular function (gene ontology) using the OMICS platform, and they corresponded to 405 proteins (14.31%) involved in organic cyclic compound binding, with 544 proteins (19.21%) having transferase activity, 586 proteins (20.7%) having hydrolase activity, 405 (14.31%) involved in heterocyclic compound binding, 242 genes (8.55%) having oxidoreductase activity, 235 (8.3%) genes having catalytic activity acting on nucleic acids, and 414 proteins (11.29%) having catalytic activity and acting on a protein (Figure 1A).

Proteins were classified considering their function in biological processes, and they corresponded to 308 proteins (1.63%) involved in macromolecule localization, 573 proteins (3.03%) involved in the regulation of metabolic processes, 1917 proteins (10.14%) involved in cellular metabolic processes, 1763 proteins (9.33%) involved in primary metabolic processes, 581 proteins (3.07%) involved in the establishment of localization, 298 proteins (1.58%) involved in postembryonic development, 377 proteins (2%) involved in cell communication, 900 proteins (4.76%) involved in biosynthetic processes, 324 proteins (1.71%) involved in positive regulation of cellular processes, 1630 proteins (8.63%) involved in nitrogen compound metabolic process, 698 proteins (3.69%) involved in response to chemicals, 328 proteins (1.74%) involved in response to external stimulus, 702 proteins (3.71%) involved cellular response to stimulus, 963 proteins (5.1%) acting on cellular component organization or biogenesis, 383 proteins (2.02%) involved in catabolic processes, 308 proteins (1.63%) involved in signal transduction, 325 proteins (1.72%) involved developmental process concerning reproduction, 290 proteins (1.53%) involved in regulation of biological quality, 454 proteins (2.4%) involved in response to biological stimulus, 1.891 proteins (10.01%) involved in organic substance metabolic processes, 815 proteins (4.31%) involved the regulation of cellular processes, 577 proteins involved in multicellular organism development, 706 proteins (3.74%) involved in response to stress, 361 proteins (1.91%) involved in cellular localization, 465 proteins (2.46%) involved in small molecule metabolic processes, 675 proteins (3.57%) involved in anatomical structure development, and 287 proteins (1.52%) involved in response to endogenous stimulus (Figure 1B).

### 2.3. U. compressa Encodes Antioxidant Enzymes, Enzymes for ASC and GSH Synthesis, and Metallothioneins

The genome of *U. compressa* encodes three SOD, 11 AP, no CAT, two DHAR, two GR, three GP, and ten PRX (Table S1). In addition, 28 thioredoxins (TRXs) that reduce PRXs and five NADPH-dependent thioredoxin reductases (TRRs) that reduce TRXs were identified (data not shown). Antioxidant enzymes showed similarity to plant, green microalgae, bacteria, and oomycete enzymes. In this sense, the 11 genes encoding AP bind reads of transcriptomes previously obtained in *U. compressa*, ranging from 521,621 to eight reads, indicating that all AP genes are expressed in *U. compressa* (data not shown). In addition, the lack of CAT genes has been corroborated by the absence of CAT activity in vitro using protein extracts [14]. The genome encodes one L-galactonolactone dehydrogenase (L-GLDH) and one L-galactose dehydrogenase (L-GDH), the last enzymes for ASC synthesis, and three GCS and one GS, which synthesize GSH, showing similarity to plant enzymes (Table S1). Five copies of the metallothionein UcMT1 were identified (data not shown), but genes encoding UcMT2 and UcMT3 were absent. In addition, genes encoding phytochelatin synthase were not identified, even though PCs are synthesized in response to copper stress [19,22], indicating that the *U. compressa* genome is not yet complete.

In contrast, the genome in the brown filamentous alga *E. siliculosus* showed six SOD, eleven CAT, one AP, one DHAR, two GR, and six GP [2]. In this sense, *U. compressa* has shown a higher tolerance to copper (10 µM) than *E. siliculosus* (2.5 µM), and the former exhibited a strong AP activity in response to copper stress [34]. The high tolerance of *U. compressa* to copper stress is explained, at least in part, by the high number of AP genes that are expressed in *U. compressa* compared to the single AP gene in *E. siliculosus*. On the other hand, *E. siliculosus* can accumulate copper in its tissue, but in lower amounts than *U. compressa*, since *E. siliculosus* cultivated with 2.5 µM of copper for 10 d accumulated 1.8 µg g-1 of dry tissue (DT), and *U. compressa* cultivated with 2.5 µM of copper for 10 d accumulated 100 µg g-1 of DT [18,34]. In this sense, the *E. siliculosus* genome encodes only two MTs, whereas the *U. compressa* genome encodes at least five copies of UcMT1, and probably several copies of UcMT2 and UcMT3. The higher number of UcMT genes found in the *U. compressa* genome may explain the higher accumulation of copper in this alga compared to other algae.

### 2.4. U. compressa Encodes Signal Transduction Protein Kinases

**Figure 2:**
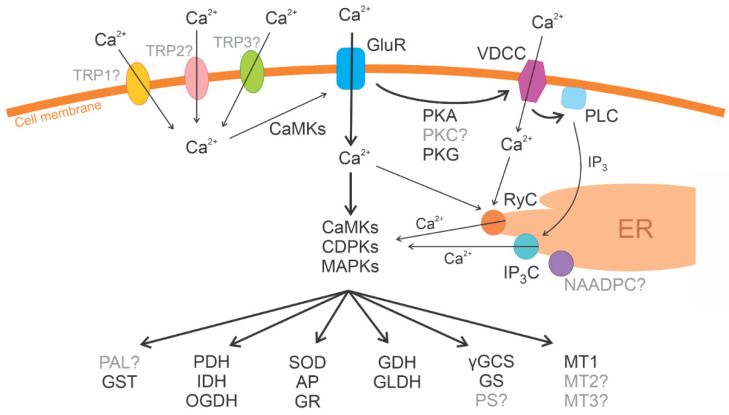
Scheme of Protein-coding genes in the *U. compressa* genome: superoxide dismutase (SOD), ascorbate peroxidase (AP), glutathione reductase (GR), and peroxiredoxin (PRX); L-galactono-lactone dehydrogenase (L-GLDH), L-galactose dehydrogenase, glutamate-cysteine synthase (GCS), and glutathione synthase (GS); metallothioneins UcMT1, UcMT2, and UcMT3; phenylalanine ammonia lyase (PAL); pyruvate dehydrogenase (PDH), isocitrate dehydrogenase (IDH), and 2-oxoglutarate dehydrogenase (OGDH); mitogen-activated protein kinases (MAPKs); calcium-dependent protein kinases (CDPKs); calcineurin B-like protein kinases (CBLPKs) and calcium/calmodulin protein kinases (CaMKs); transient receptor potential (TRP) channels, voltage-dependent calcium channels (VDCCs), phospholipase D (PLD), calcium/ryanodine-dependent channel (RyC), IP3-dependent channel (IP3C), NAADP-dependent channel (NAADPC), and ionotropic glutamate receptors (GluR); cAMP-dependent protein kinase (PKA) and cGMP-dependent protein kinase (PKG). Those proteins that have been identified in the *U. compressa* genome and have also been experimentally detected are depicted in black, and those that have been experimentally detected and are not present in the *U. compressa* genome are depicted in gray with a question mark.

The *U. compressa* genome encodes eight MAPKs, two MAPKKs, and 19 MAPKKKs, and they showed sequence similarity to plant, green microalgae, and bacterial protein kinases (PKs) (Table S2). Thus, the genome of *U. compressa* encodes 29 MAPKs. The genome encodes 10 CDPKs, 18 CBLPKs (plant CIPKs), and 14 CaMKs, showing similarity to plant, green microalgae, and bacterial PKs. Two PKA and three PKG genes were also identified, and they showed similarity to green microalgae PKs (Table S2). Thus, *U. compressa* genome encodes 76 PKs that have been experimentally predicted using the inhibitors of animal PKs [21,24] (Figure 2).

It has been recently shown that 48 different MAPKs are present in the genomes of green microalgae, such as *Ostreococcus*, *Micromonas*, *Bathycoccus*, *Chlamydomonas*, *Volvox*, *Gonium*, *Chlorella*, and others, and they were classified in four groups [35]. In fact, the genome of the unicellular green microalga *C. reinhardtii* encodes 17 MAPKs, 2 MAPKKs, and 108 MAPKKKs [36], which is in accord with findings in the multicellular *U. compressa*, except for the larger number of MAPKKKs found in *C. reinhardtii*. In addition, 15 genes encoding CDPKs were identified in the genome of *C. reinhardtii* [37], which is in accord with those present in the genome of *U. compressa*.

### 2.5. Ethylene- and ABA-Responsive Regulatory Transcription Factors and Other Plant-like Transcription Factors

Subunits of general transcription factors (GTFs) were found in the *U. compressa* genome, such as subunits of TFIIA, TBIIB, TFIIE, and TFIIH, which are related to RNA polymerase II; subunits of TFIIIB and TFIIIC, which are related to RNA Polymerase III; and five subunits of the SWI/SNF remodeling complex (data not shown). Regulatory transcription factors (RTFs) were also encoded, corresponding to 14 Ehylene-responsive factors (ERF/AP-2), 14 MYB-like, 11 abscisic acid (ABA)-responsive factors, 6 basic helix–loop–helix (bHLH), 4 GTE-like, 3 nuclear factor Y (NFY), 2 GATA-like, 1 heat shock transcription factor (HSTF), 1 WRKY, and others, and they showed similarity to plant and green microalgae RTFs (Table S3).

The most interesting fact is the high number ethylene- and ABA-responsive transcription factors (TFs) that were found, indicating that these two environmental stimuli-responsive hormones play important roles in *U. compressa*. In addition, *U. mutabilis* showed 15 genes of ethylene-responsive TFs and five genes of ABA-responsive TFs, indicating that ethylene and ABA also play important roles in *U. mutabilis* [11]. The *U. mutabilis* genome encodes five MYB-like, three bHLH, two GATA-like, two HSTF, two WRKY, and other families of TFs that are not found in *U. compressa* (Table S3), as well as 32 subunits of SWI/SNF remodeling complex. These results indicate that the number of RTFS is more diverse in *U. mutabilis* than in *U. compressa*, which is in accord with its more complex shape and larger blade size.

### 2.6. U. compressa Encodes Enzymes for ACC and ABA-Aldehyde Synthesis but Lacks Enzymes Involved in Ethylene and ABA Synthesis

Two genes encoding S-adenosyl methionine (SAM) synthase, which synthesizes SAM, and two genes encoding ACC synthase, which synthesizes 1-aminocyclopropane carboxylic acid (ACC), were found in the *U. compressa* genome, but the gene that encodes ACC oxidase, which synthesizes ethylene, was not identified (Table S4). Two genes encoding 9-epoxicarotenoid dioxygenase, one gene encoding alcohol dehydrogenase, and one gene encoding zeaxanthin epoxidase were detected, but the gene that encodes ABA-aldehyde oxidase, which synthesizes abscisic acid (ABA), was not found (Table S4). Thus, ethylene and ABA may not be synthesized in *U. compressa*, suggesting that ACC and ABA-aldehyde may act as hormones in *U. compressa*. In this sense, it has been shown that ACC, and not ethylene, is essential for embryo viability in plants, and it can act as a signaling molecule [38].

Regarding auxin synthesis, the genome of *U. compressa* did not encode tryptophan aminotransferase or YUCCA-like flavin monooxygenase, suggesting that 3-indoleactic acid is not synthesized in *U. compressa*. In addition, genes encoding enzymes involved in the synthesis of gibberellins, cytokinins, salicylic acid (SA), and jasmonic acid (JA) were not detected in the *U. compressa* genome. Genes encoding isochorismate synthase (ICS) and phenylalanine ammonia lyase (PAL), enzymes that synthesize SA, were not found in the genome. Surprisingly, PAL activity has been detected in protein extracts of *U. compressa* [20], suggesting that the genome is not complete, or that the amino acid sequence of PAL in *U. compressa* is highly divergent from that of PAL in plants (Figure 2). In addition, five arachidonic acid-dependent lipoxygenase (LOX) genes, but no allene oxide synthase (AOS) or allene oxidase cyclase (AOC) genes, were detected in the *U. compressa* genome, indicating that JA is not synthesized in *U. compressa*. It is important to point out that LOX activity in *U. compressa* is dependent on arachidonic acid as a substrate, as in animals, and not on linoleic/linolenic acids, as in plants [20].

In contrast, the genome of *U. mutabilis* encodes ABA-aldehyde oxidase, suggesting that ABA is synthesized in *U. mutabilis*. In addition, the *U. mutabilis* genome encodes enzyme ICS, but lacks PAL [11]. In fact, hormones were quantified in *U. mutabilis*, showing that there is a high level of the auxin indole-acetic acid and SA; a low level of ethylene and ABA; traces of gibberellins; and no JA, brassinosteroids, or strigolactones. In the future, it will be interesting to analyze whether SA, JA, auxin, and gibberellins can be detected in *U. compressa*.

### 2.7. U. compressa Genome Encodes Proteins Involved in Cell Cycle Regulation

In plants, the cell cycle is regulated by cyclins, cyclin-dependent PKs (CDKs), E2F and retinoblastoma (RB) TFs, and Wee kinase [39]. The genome of *U. compressa* encodes eight cyclins corresponding to one cyclin A, two cyclin B, one cyclin C, one cyclin H, one cyclin L, one cyclin P, and one cyclin S, with similarity to plant cyclins, and lacks cyclin D. In addition, it encodes twenty-four CDKs, including CDKA, CDKB, CDKC, CDKD, CDKF, and CDKG, similar to plant CDKs, and one CDK1, two CDK2, one CDK7, and two CDK12, similar to fungal and animal CDKs (Table S5). Genes encoding E2F, RB, and Wee kinase were not identified in the *U. compressa* genome.

The latter is in accord with cyclins in the *U. mutabilis* genome, which encodes nine cyclins corresponding to four cyclin A, one cyclin B, and one each of cyclin E, F, G, and H, similar to plant cyclins, and lacks cyclin D [11]. In contrast, CDKs that showed similarity to fungal and animal cyclins were not found in the *U. mutabilis* genome. Thus, CDKs in *U. mutabilis* showed higher similarity to plant CDKs than those of *U. compressa*.

### 2.8. U. compressa Genome Encodes Enzymes and Transporters for Cell Wall Synthesis, and Animal Extracellular Matrix Proteins

The cell wall of green macroalgae contains ulvan polysaccharides, which are constituted by sulfated xylose, sulfated rhamnose, iduronic acid, and glucuronic acid [40]. Green algae cell walls also contain cellulose, pectin, and hydroxyproline-rich glycoproteins [41]. Seven cellulose synthases (CSs) were found in the *U. compressa* genome, showing similarity to plant and bacterial CSs (Table S6). In addition, genes encoding eight hydroxyprolyl O-arabinosyl transferases, two glucomannan-mannosyl transferase, one UDP-glucosamine transferase, two UDP-rhamnose transporters, two UDP-uronic acid transporters, and one pectin lyase were identified, having similarity to plant and green microalgae proteins (Table S6). Surprisingly, genes encoding four collagens, three extensins, one expansin, and two elastins were detected, having similarity to animal extracellular matrix (ECM) proteins (Table S6). In addition, four fibronectin genes were encoded, having similarity to bacteria and green microalgae proteins (Table S6). Thus, the cell wall of *U. compressa* might present a combination of plant cell wall and animal ECM, and animal ECM-coding genes may have been lost during the evolution of land plants.

The genome of *U. mutablis* encodes 6 CSs, 11 glycosyl transferases, 16 extensins, and 1 expansin [11]. In addition, it has been reported that *U. mutabilis* encodes 28 collagen genes, but no fibronectin or elastin genes were described [11]. Therefore, the cell wall of Ulvophyceae may be a mixture of plant cell wall and animal ECM.

### 2.9. U. compressa Genome Encodes Channels for Extracellular Calcium Entry and Intracellular Calcium Release

The *U. compressa* genome encodes three VDCCs with similarity to *C. reinhardtii* VDCCs, three calcium/ryanodine-dependent channels with similarity to fungal and animal channels, and two IP3-dependent channels with similarity to plant channels (Table S7). Surprisingly, genes encoding TRP channels were not identified in the *U. compressa* genome even though their existence has been experimentally demonstrated using the inhibitors of human TRPs [27,28]. Eleven ionotropic glutamate receptor (GluR) genes were identified in the *U. compressa* genome, and they showed similarity to plant, green microalgae, and animal receptors (Figure 2). Surprisingly, serotonin- and dopamine-like receptors were not found in the alga genome, even though they have been experimentally detected in *U. compressa* [29].

The genome of *E. Siliculosus* encodes 18 TRP channels [2], which are functional TRPs that allow extracellular calcium entry [42]. The genome of *C. reinhardtii* encodes 10 TRP channels and they are functional mosaic TRPs, as in *U. compressa*, since they are a combination of human TRPA, TRPC, TRPM, and TRPV [43]. Thus, TRP channels exist in marine macroalgae and in green microalgae, even though they are absent in plant genomes, indicating that TRP genes may have been lost during the evolution of land plants. It has been shown that the brown alga *E. siliculosus*, subjected to copper stress, also releases amino acids and noradrenalin to the extracellular medium, which may activate glutamate- and noradrenalin-like receptors (M. Gómez, unpublished). It was determined that the genome of *E. siliculosus* encodes two ionotropic GluR in [2], indicating that released amino acids may activate these receptors. Serotonin-, adrenalin-, or noradrenalin-like receptors have not been identified in the *E. siliculosus* genome [2].

Protein-coding genes previously detected in the genome of *U. compressa* (this work) mainly encode proteins with similarity to those of plant and green microalgae (74.4%), but also to animal, bacterial, and fungal proteins (25.6%). In this sense, it has been recently shown that the genome of unicellular green microalga *Spiroglea muscicola* and *Mesotaenium endlicheriarim*, which share a habitat with terrestrial bryophytes (moss), encodes proteins involved in ABA perception and signaling involved in desiccation tolerance and that these genes have been acquired by horizontal gene transfer (HGT) from soil bacteria [44]. In addition, it has been shown that the genome of the moss *Physcomitrella patens* encodes genes that have been acquired from bacteria and fungi by HGT [45]. Thus, genes in U. compressa that showed similarity to bacterial and fungal genes may have been transferred from marine bacteria and fungi to U. compressa genome by HGT.

In conclusion, the genome of *U. compressa* encodes antioxidant enzymes such as SOD, AP, DHAR, GR, and PRX, but not CAT, and enzymes that synthesize ASC and GSH showed similarity to plants, green microalgae, and bacterial antioxidant enzymes. Several MAPKs, CDPKs, CBLPKs, and CaMKs involved in signal transduction are encoded in the *U. compressa* genome, and they showed similarity to plant, green microalgae, and bacterial PKs. Several ethylene- and ABA-dependent RTFs are encoded, and they showed similarity to plant and green microalgae RTFs. Surprisingly, the genes, encoding the last enzymes for ethylene and ABA syntheses, were not found, suggesting that ABA and ethylene are not synthesized in *U. compressa* and that ACC and ABA-aldehyde may act as signaling molecules. Enzymes involved in auxin, gibberellins, cytokinins, brassinosteroids, strigolactones, and SA and JA syntheses were not found in the *U. compressa* genome. Cyclins A, B, C, H, L, P, and S (but not cyclin D); CDKs A, B, C, F, and G; and CDKs 2, 7, and 12 are encoded in the *U. compressa* genome, and they showed similarity to plant animal and fungal CDKs. Genes that encode CSs were identified, and they showed similarity to plant, green microalgae, and bacterial CSs. Transporters of sugars for ulvan synthesis were detected, similar to plant transporters, and proteins of animal ECM, such as collagen, elastin, extensins, and expansins, similar to animal proteins, were also identified. In addition, the *U. compressa* genome encodes few VDCCs, allowing extracellular calcium entry which showed similarity to *C. reinhardtii* VDCCs; calcium/ryanodine-dependent channels, which allow intracellular calcium release from ER, similar to animal channels; IP3-dependent calcium channels, showing similarity to plant channels; and ionotropic GluR, displaying similarity to plant, green microalgae, and animal GluR. In this work, TRP channels were not identified in the *U. compressa* genome even though they have been experimentally detected, indicating that the genome is not yet complete (Figure 2). Thus, *U. compressa* protein-coding genes have similarity to plant and green microalgae genes (74.4%), but also to animal, bacterial, and fungal genes (25.6%). In the future, the missing part of the *U. compressa* genome could be completed using PacBio HiFi sequencing.

## 3. Materials and Methods

### 3.1. DNA Purification and Sequencing

The algae were collected in Cachagua (32°34′47″ S 71°27′11″ O), a pristine site of central Chile, transported to the laboratory in a cooler with ice, manually cleaned in artificial seawater, and sonicated three times for 2 min using an ultrasound bath. The sonicated algae did not show bacteria bind on the cell wall under electron microscopy [17].

Total DNA from *U. compressa* was purified using the method described by [46]. Briefly, 0.1 g of fresh tissue (FT) was frozen with liquid nitrogen and homogenized in a mortar using a pestle. One mL of buffer containing 100 mM of Tris-HCL, 20 mM EDTA, 1% N-lauryl sarcosine, 2% polyvinyl pyrrolidone, 0.2% of β-mercaptoethanol, and 1.5 M NaCl was added, and the homogenization was pursued until thawing. The homogenate was centrifuged at 14,200× *g* for 10 min at 4 °C. The supernatant was recovered and 1/9 volume of absolute ethanol, ¼ volume of potassium acetate, and 1 volume of chloroform/isoamylic alcohol (24:1 *v*/*v*) were added; the solution was then incubated at −20 °C for 20 min. The suspension was centrifuged at 14,200× *g* for 20 min at 4 °C and the upper aqueous phase was recovered. In total, 100 μg of RNAse A (Sigma-Aldrich, Saint Louis, MO, USA) was added, and the solution was incubated at 37 °C for 30 min. One volume of chloroform/isoamylic alcohol (24:1) was added, and the suspension was incubated at −20 °C for 20 min. The suspension was centrifuged at 14.200× *g* for 20 min at 4 °C; the upper aqueous phase was recovered; 0.8 volume of isopropanol, 0.1 volume of 3 M sodium acetate, and 0.2% β-mercaptoethanol were added; and the suspension was incubated at −20 °C for 1 h. The suspension was centrifuged at 14,200× *g* for 20 min at 4 °C and the supernatant was discarded. The pellet (total DNA) was washed with 1 mL of ethanol 70% and the suspension was centrifuged at 14.200× *g* for 20 min. The pellet was dried at room temperature in a hood and solubilized in 50 μL of nuclease-free water. Total DNA was sent to Novogen (Sacramento, CA, USA) and sequenced at BGI (Shenzhen, China) using PacBio and pair-ended Illumina techniques.

### 3.2. Cleaning of the Reads

PacBio reads were cleaned from bacterial sequences using MINIMAP2 software [47] and the bacterial database Prokaryotic RefSeq Genomes from NCBI. Illumina reads were cleaned for quality using PRINSEQ software [48] and cleaned from bacterial sequences using BOWTIE v.2.2 software [49] and a library that was prepared with 3200 bacterial genomes.

### 3.3. Assembly of the Genome and Annotation of Genes

PacBio contigs and Illumina reads were assembled using MaSuRCA v4.0.8 [49]; scaffolds were prepared using LONGSTITCH v1.0.1 software [50], polished using POLCA software [51], and reordered using RAGTAG v2.1.0 software [52] with the assembled genome of *U. mutabilis* as a reference [27]. Novek repeated sequences were identified using MAKER, and known repeated sequences were detected using REPEATMASKER software and REPBASE database [53]. Structural RNAs were detected using StructRNAfinder software [54]. The annotation of protein-coding genes was performed using MAKER v3.01.03 software [55], which uses an *ab initio* system for gene finding. To check that predicted genes were true genes, the reads of previously obtained transcriptomes of *U. compressa* [30,31] were mapped to protein-coding genes in the *U. compressa* genome using STAR software v.2. 7.10A [56]. Core protein-coding genes were identified using BUSCO software [57].

### 3.4. Functional Classification of Genes

Protein-coding genes were classified according to gene ontology using OMICS platform [58,59], and those having an e-value < e^−3^ were selected. Protein-coding genes were classified according to GO domain, corresponding to molecular function and biological process using NR database. Protein-coding genes encoding proteins and enzymes of the antioxidant system, transduction signal PKs, transcription factors, proteins for cell cycle regulation, proteins for cell wall synthesis, and proteins for extracellular calcium entry and intracellular calcium release were curated by hand from MAKER-blasted annotated genes using an e-value < e^−3^, the same e-value used in previous works [30,31].

## Figures and Tables

**Table 1:** Summary statistics of *U. compressa* genome and comparison with *U. mutabilis*.

Genome	*U. compressa*	*U. mutabilis*
Genome size	80.8 Mb	98.5 Mb
Number of scaffolds	2601	318
Scaffolds N50	0.46 Mb	0.6 Mb
Scaffolds L50	48	46
Percentage of GC content	57.3%	57.2%
Number of protein coding genes	19,207	12,924
Gene density	238 genes/Mb	131 genes/Mb
Average intron per gene	3.6	nd
Average exon length	322 bp	nd
Average intron length	479 bp	nd
Number of exons in CDS	88,404	nd
Number of introns in CDS	69,134	nd
Percentage of core genes	80%	92%
Repetitive elements (RE)	18.9%	35%
LINE	1.77%	15.1%
LTR	1.52%	9.4%
DNA elements	0.61%	
Unknown RE	15.04%	10.5%
Number of rRNAs	18	nd
Number of tRNAs	109	nd
Number of snoRNAs	27	nd
Number of snRNAs	11	nd
Number of primary miRNAs	87	nd

nd: not determined.

## Data Availability

Data is available in the NCBI database as Bioproject PRJNA82527.

## Supplementary Materials

The following supporting information can be downloaded at: https://www.mdpi.com/article/10.3390/ijms23137279/s1.

## Abbreviations

ABA	abscisic acid
ACC	1-aminocyclopropane carboxylic acid
AP	ascorbate peroxidase
AOC	allene oxide cyclase
AOS	allene oxide synthase
ASC	ascorbate
CAT	catalase
CaMK	calcium/calmodulin-dependent protein kinase
CBLPK	calcineurin b-like protein kinase
CDK	cyclin-dependent protein kinase
CDPK	calcium-dependent protein kinase
CS	cellulose synthase
ECM	extracellular matrix
GCS	glutamyl-cysteinyl synthase
GluR	glutamate receptor
GS	glutathione synthase
GSH	glutathione
GR	glutathione reductase
ICS	isochorismate synthase
L-GLDH	L-gulonolactone dehydrogenase
L-GDH	L-galactose dehydrogenase
LOX	lipoxygenase
MAPK	mitogen-activated protein kinase
MT	metallothionein
PAL	phenylalanine ammonia lyase
PCs	phytochelatins
PKA	cAMP-dependent protein kinase
PKG	cGMP-dependent protein kinase
PRX	peroxiredoxin
SAM	S-adenosyl methionine
TRP	transient receptor potential channel
TRR	NADPH-dependent thioredoxin reductase
TRX	thioredoxin
VDCC	voltage-dependent calcium channels
