# Supplementary material for: The Genome of the Marine Alga Ulva compressa (Chlorophyta) Reveals Protein-Coding Genes with Similarity to Plants and Green Microalgae, but Also to Animal, Bacterial, and Fungal Genes
Source: Int J Mol Sci. 2022 Jun 30;23(13):7279. doi: 10.3390/ijms23137279 (PMC9266709; doi:10.3390/ijms23137279)
Supplement: Supplementary file 1 [file ijms-23-07279-s001.zip › ijms-1776681-supplementary.pdf]

Table S1. Protein-coding genes of antioxidant enzymes, and enzymes involved in ASC and GSH synthesis in *U. compressa*

**Gene**	**Annotation**	**Best blast hit**
ucom_00009177-RA	Superoxide dismutase	*Prunus persica* (Plant)
ucom_00015047-RA	Superoxide dismutase	*Chlorella vulgaris* (Green microalgae)
ucom_00015048-RA	Superoxide dismutase	*Nostoc flagelliforme* (Cyanobacteria)
ucom_00015296-RA	Ascorbate peroxidase	*Brassica oleracea* (Plant)
ucom_00008899-RA	Ascorbate peroxidase	*Prunus avium* (Plant)
ucom_00013441-RA	Ascorbate peroxidase	*Brassica oleracea* (Plant)
ucom_00000286-RA	Ascorbate peroxidase	*Hevea brasiliensis* (Plant)
ucom_00001559-RA	Ascorbate peroxidase	*Volvox reticuliferus* (Green microalga)
ucom_00010335-RA	Ascorbate peroxidase	*Chlorella sorokiniana* (Green microalga)
ucom_00019150-RA	Ascorbate peroxidase	*Phytophthora cactorum* (Oomycete)
ucom_00010831-RA	Ascorbate peroxidase	*Phytophthora fragariae* (Oomycete)
ucom_00019192-RA	Ascorbate peroxidase	*Phytophthora fragariae* (Oomycete)
ucom_00018857-RA	Ascorbate peroxidase	*Phytophthora fragariae* (Oomycete)
ucom_00018785-RA	Ascorbate peroxidase	*Vibrio parahaemolyticus* (Bacteria)
ucom_00013667-RA	Dehydroascorbate reductase	*Solanum lycopersicum* (Plant)
ucom_00013666-RA	Dehydroascorbate reductase	*Solanum lycopersicum* (Plant)
ucom_00006276-RA	Glutathione reductase	*Salvia splendens* (Plant)
ucom_00003402-RA	Glutathione reductase	*Solanum chilense* (Plant)
ucom_00002512-RA	Glutathione peroxidase	*Zea mays* (Plant)
ucom_00012289-RA	Glutathione peroxidase	*Lapillicoccus jejuensis* (Bacteria)
ucom_00001838-RA	Glutathione peroxidase	*Geobacillus genomosp* (Bacteria)
ucom_00002178-RA	Peroxiredoxin	*Gossypium barbadense* (Plant)
ucom_00006242-RA	Peroxiredoxin	*Chlorella vulgaris* (Green microalga)
ucom_00010225-RA	Peroxiredoxin	*Volvox reticuliferus* (Green microalga)
ucom_00006241-RA	Peroxiredoxin	*Chlorella vulgaris* (Green microalga)
ucom_00007955-RA	Peroxiredoxin	*Cyanobacterium sp.* (Cyanobacteria)
ucom_00000755-RA	Peroxiredoxin	*Raphidocelis sp.* (Green Microalga)
ucom_00005297-RA	Peroxiredoxin	*Helicoverpa armigera* (Insect)
ucom_00003553-RA	Peroxiredoxin	*Leptospira interrogans* (Bacteria)
ucom_00011880-RA	Peroxiredoxin	*Chloroflexi bacterium* (Bacteria)
ucom_00004014-RA	Peroxiredoxin	*Vibrio parahaemolyticus* (Bacteria)
ucom_00005052-RA	L-galactono-lactone dehydrogenase	*Brassica olerace* (Plant)
ucom_00011588-RA	L-galactose dehydrogenase	*Selaginella moellendorffii* (Plant)
ucom_00001065-RA	Glutamate-cysteine synthase	*Arabidopsis thaliana* (Plant)
ucom_00001067-RA	Glutamate-cysteine synthase	*Camelina sativa* (Plant)
ucom_00001068-RA	Glutamate-cysteine synthase	*Arabidopsis thaliana* (Plant)
ucom_00011402-RA	Glutathione synthase	*Solanum chilense* (Plant)

Table S2. Protein-coding genes involved in signal transduction in *U. compressa*

Gene	Annotation	Best blast hit
ucom_00005489-RA	MAPK1	*Paramecium primaurelia* (Ciliophora)
ucom_00014789-RA	MAPK3	*Raphidocelis subcapitata* (Green microalga)
ucom_00005113-RA	MAPK4	*Naegleria fowleri* (Excavata)
ucom_00002620-RA	MAPK5	*Brassica oleracea* (Plant)
ucom_00014385-RA	MAPK8	*Raphidocelis subcapitata* (Green microalga)
ucom_00000788-RA	MAPK9	*Prunus avium* (Plant)
ucom_00007327-RA	MAPK12	*Mycobacterium simiae* (Bacteria)
ucom_00003193-RA	MAPK14	*Triticum aestivum* (Plant)
ucom_00004185-RA	MAPKK	*Volvox reticuliferus* (Green microalga)
ucom_00008671-RA	MAPKK	*Panicum virgatum* (Plant)
ucom_00004102-RA	MAPKKK	*Volvox reticuliferus* (Green microalga)
ucom_00018435-RA	MAPKKK3	*Streptomyces sp.* (Bacteria)
ucom_00018435-RA	MAPKKK3	*Frankia irregularis* (Bacteria)
ucom_00013498-RA	MAPKKK6	*Tetrabaena socialis* (Green microalgae)
ucom_00001835-RA	MAPKKK10	*Panicum hallii* (Plant)
ucom_00010886-RA	MAPKKK10	*Volvox reticuliferus* (Green microalga)
ucom_00014486-RA	MAPKKK10	*Phytophthora cinnamomi* (Oomycete)
ucom_00006679-RA	MAPKKK10	*Volvox reticuliferus* (Green microalga)
ucom_00010433-RA	MAPKKK11	*Volvox reticuliferus* (Green microalga)
ucom_00012934-RA	MAPKKK11	*Volvox reticuliferus* (Green microalga)
ucom_00012529-RA	MAPKKK11	*Volvox reticuliferus* (Green microalga)
ucom_00010433-RA	MAPKKK11	*Volvox reticuliferus* (Green microalga)
ucom_00012934-RA	MAPKKK11	*Volvox reticuliferus* (Green microalga)
ucom_00012529-RA	MAPKKK11	*Volvox reticuliferus* (Green microalga)
ucom_00003193-RA	MAPKKK18	*Triticum aestivum* (Plant)
ucom_00005329-RA	MAPKKK20	*Planctomycetes bacterium* (Bacteria)
ucom_00001653-RA	MAPKKK21	*Auxenochlorella prototech* (Green microalga)
ucom_00005320-RA	MAPKKK21	*Volvox reticuliferus* (Green microalga)
ucom_00001653-RA	MAPKKK21	*Auxenochlorella protothecoides* (Green microalgae)
ucom_00005688-RA	CDPK1	*Brassica carinata* (Plant)
ucom_00008916-RA	CDPK2	*Nicotiana tabacum* (Plant)
ucom_00013388-RA	CDPK4	*Solanum commersonii* (Plant)
ucom_00014013-RA	CDPK12	*Triticum aestivum* (Plant)
ucom_00018169-RA	CDPK15	*Solanum commersonii* (Plant)
ucom_00001967-RA	CDPK17	*Arabidopsis thaliana* (Plant)
ucom_00005689-RA	CDPK17	*Arabidopsis thaliana* (Plant)
ucom_00001561-RA	CDPK19	*Chlamydomonas reinhardtii* (Green microalga)
ucom_00011484-RA	CDPK23	*Solanum commersonii* (Plant)
ucom_00011733-RA	CDPK27	*Solanum commersonii* (Plant)
ucom_00015115-RA	CIPK1	*Volvox reticuliferus* (Green microalga)
ucom_00017140-RA	CIPK2	*Chlamydomonas sp* (Green microalga)
ucom_00010939-RA	CIPK2	*Volvox reticuliferus* (Green microalga)
ucom_00002198-RA	CIPK3	*Nostoc sp.* (Cyanobacteria)
ucom_00002987-RA	CIPK3	*Micromonospora sp.* (Bacteria)
ucom_00005316-RA	CIPK5	*Oryza sativa* (Plant)
ucom_00010134-RA	CIPK5	*Chlamydomonas sp* (Green microalga)
ucom_00012659-RA	CIPK5	*Volvox reticuliferus* (Green microalga)
ucom_00018135-RA	CIPK5	*Verrucomicrobia* (Bacteria)
ucom_00007971-RA	CIPK6	*Verticillium longisporum* (Fungus)
ucom_00013170-RA	CIPK8	*Raphidocelis subcapitata* (Green microalga)

ucom_00011616-RA	CIPK9	*Oryza sativa* (Plant)
ucom_00004189-RA	CIPK10	*Volvox reticuliferus* (Green microalga)
ucom_00016841-RA	CIPK13	*Verrucomicrobia* (Bacteria)
ucom_00011612-RA	CIPK13	*Verrucomicrobia* (Bacteria)
ucom_00003198-RA	CIPK17	*Oryza sativa* (Plant)
ucom_00011614-RA	CIPK25	*Verrucomicrobia* (Bacteria)
ucom_00010938-RA	CIPK30	*Volvox reticuliferus* (Green microalga)
ucom_00002341-RA	CaMK	*Raphidocelis subcapitata* (Green microalga)
ucom_00002839-RA	CaMK	*Glycine soja* (Plant)
ucom_00005349-RA	CaMK	*Coccomyxa sp.* (Green microalga)
ucom_00005475-RA	CaMK	*Volvox reticuliferus* (Green microalga)
ucom_00007621-RA	CaMK	*Coccomyxa sp.* (Green microalga)
ucom_00009041-RA	CaMK	*Volvox reticuliferus* (Green microalga)
ucom_00009668-RA	CaMK	*Coccomyxa sp.* (Green microalga)
ucom_00009925-RA	CaMK	*Coccomyxa sp.* (Green microalga)
ucom_00010519-RA	CaMK	*Trebouxia sp.* . (Green microalga)
ucom_00013170-RA	CaMK	*Raphidocelis subcapitata* (Green microalga)
ucom_00015152-RA	CaMK	*Ulva partita* (Green Macroalga)
ucom_00015869-RA	CaMK	*Coccomyxa sp.* (Green microalga)
ucom_00016998-RA	CaMK	*Ulva partita* (Green Macroalga)
ucom_00017140-RA	CaMK	*Raphidocelis subcapitata* (Green microalga)
ucom_00001613-RA	PKA	*Scenedesmus sp.* (Green microalga)
ucom_00010638-RA	PKA	*Trebouxia sp.* (Green microalga)
ucom_00009499-RA	PKG	*Chloropicon primus* (Green microalga)
ucom_00003150-RA	PKG	*Haematococcus lacustris* (Green miroalga)
ucom_00003295-RA	PKG	*Coccomyxa subellipsoidea* (Green microalga)

Table S3. Protein-coding genes of regulatory transcription factors in *U. compressa*

**Gene**	**Annotation**	**Best blast hit**
ucom_00007866-RA	Ethylene-responsive factor	*Gossypium tomentosum* (Plant)
ucom_00007976-RA	Ethylene-responsive factor	*Sorghum bicolor* (Plant)
ucom_00012589-RA	Ethylene-responsive factor	*Triticum carthlicum* (Plant)
ucom_00005095-RA	Ethylene-responsive factor	*Triticum carthlicum* (Plant)
ucom_00008969-RA	Ethylene-responsive factor	*Triticum carthlicum* (Plant)
ucom_00007276-RA	Ethylene-responsive factor	*Arabidopsis thaliana* (Plant)
ucom_00011467-RA	Ethylene-responsive factor	*Arabidopsis thaliana* (Plant)
ucom_00011723-RA	Ethylene-responsive factor	*Triticum carthlicum* (Plant)
ucom_00004069-RA	Ethylene-responsive factor	*Macleaya cordata* (Plant)
ucom_00004084-RA	Ethylene-responsive factor	*Gossypium hirsutum* (Plant)
ucom_00001469-RA	Ethylene-responsive factor	*Arabidopsis thaliana* (Plant)
ucom_00005424-RA	Ethylene-responsive factor	*Vanilla planifolia* (Plant)
ucom_00008223-RA	Ethylene-responsive factor	*Brassica cretica* (Plant)
ucom_00002704-RA	Ethylene-responsive factor	*Volvox sp.* (Green microalga)
ucom_00010896-RA	Myb-like	*Glycine soja* (Plant)
ucom_00018091-RA	Myb-like	*Ceratodon purpureus* (Plant)
ucom_00008077-RA	Myb-like	*Zingiber officinale* (Plant)
ucom_00006534-RA	Myb-like	*Citrus sinensis* (Plant)
ucom_00006601-RA	Myb-like	*Glycine max* (Plant)
ucom_00006031-RA	Myb-like	*Prunus armeniaca* (Plant)
ucom_00011393-RA	Myb-like	*Cucurbita argyrosperma* (Plant)
ucom_00000471-RA	Myb-like	*Zingiber officinale* (Plant)

ucom_00003291-RA	Myb-like	*Brassica cretica* (Plant)
ucom_00001695-RA	Myb-like	*Arabidopsis thaliana* (Plant)
ucom_00011503-RA	Myb-like	*Digitaria exilis* (Plant)
ucom_00003611-RA	Myb-like	*Gossypium hirsutum* (Plant)
ucom_00017619-RA	Myb-like	*Aureobasidium pullulans* (Fungus)
ucom_00002508-RA	Myb-like	*Athalia rosae* (Insect)
ucom_00005556-RA	ABA-response factor	*Colocasia esculenta* (Plant)
ucom_00000836-RA	ABA-response factor	*Triticum aestivum* (Plant)
ucom_00000839-RA	ABA-response factor	*Solanum tuberosum* (Plant)
ucom_00006887-RA	ABA-response factor	*Brassica rapa* (Plant)
ucom_00018811-RA	ABA-response factor	*Brassica rapa* (Plant)
ucom_00016800-RA	ABA-response factor	*Eucalyptus grandis* (Plant)
ucom_00015747-RA	ABA-response factor	*Glycine max* (Plant)
ucom_00017208-RA	ABA-response factor	*Gossypium hirsutum* (Plant)
ucom_00000843-RA	ABA-response factor	*Elaeis guineensis* (Plant)
ucom_00016343-RA	ABA-response factor	*Gossypium hirsutum* (Plant)
ucom_00000053-RA	ABA-response factor	*Cynara cardunculus* (Plant)
ucom_00008938-RA	Basic-helix-loop-helix	*Gossypium lobatum* (Plant)
ucom_00002004-RA	Basic-helix-loop-helix	*Abrus precatorius* (Plant)
ucom_00010047-RA	Basic-helix-loop-helix	*Brassica rapa* (Plant)
ucom_00005996-RA	Basic-helix-loop-helix	*Brassica rapa* (Plant)
ucom_00002019-RA	Basic-helix-loop-helix	*Trebouxia sp.* (Green microalga)
ucom_00006669-RA	Basic-helix-loop-helix	*Nannochloropsis sp.* (Green microalga)
ucom_00011535-RA	GTE-like 1	*Dioscorea cayenensis* (Plant)
ucom_00003840-RA	GTE-like 8	*Panicum hallii* (Plant)
ucom_00006009-RA	GTE-like 9	*Zingiber officinale* (Plant)
ucom_00012260-RA	GTE-like 10	*Triticum turgidum* (Plant)
ucom_00005927-RA	Nuclear factor Y2	*Vernicia fordii* (Plant)
ucom_00005177-RA	Nuclear factor Y2	*Jatropha curcas* (Plant)
ucom_00002150-RA	Nuclear factor C4	*Brassica rapa* (Plant)
ucom_00002619-RA	GATA-like	*Raphidocelis sp.* (Green microalga)
ucom_00012268-RA	HSTF	*Populus deltoides* (Plant)
ucom_00014452-RA	WRKY33	*Brassica carinata* (Plant)

Table S4. Protein-coding genes involved in phytohormone synthesis of *U. compressa*

Gene	Annotation	Best blast hit
ucom_00017123-RA	S-adenosylmethionine synthase	*Ulva partita* (Green macroalga)
ucom_00017541-RA	S-adenosylmethionine synthase	*Populus nigra* (Plant)
ucom_00013842-RA	ACC synthase	*Glycine max* (Plant)
ucom_00013843-RA	ACC synthase	*Acer yangbiense* (Plant)
ucom_00018965-RA	9-epoxicarotenoid dioxygenase	*Arabidopsis thaliana* (Plant)
ucom_00013375-RA	9-epoxicarotenoid dioxygenase	*Arabidopsis thaliana* (Plant)
ucom_00005184-RA	Alcohol dehydrogenase	*Volvox sp.* (Green microalga)
ucom_00003314-RA	Zeaxanthin epoxidase	*Chlamydomonas sp.* (Green microalga)

Table S5. Protein-coding genes involved cell cycle regulation in *U. compressa*.

Gene	Annotation	Best blast hit
ucom_00004603-RA	Cyclin-A1	*Brassica rapa* (Plant)
ucom_00011330-RA	Cyclin-B1	*Juglans regia* (Plant)
ucom_00010653-RA	Cyclin-B3	*Elaeis guineensis* (Plant)
ucom_00002573-RA	Cyclin-C1	*Zea mays* (Plant)
ucom_00005966-RA	Cyclin-H1	*Triticum dicoccoides* (Plant)
ucom_00001395-RA	Cyclin-L1	*Durio zibethinus* (Plant)
ucom_00012494-RA	Cyclin-P3	*Camellia sinensis* (Plant)
ucom_00010139-RA	Cyclin-S13	*Glycine soja* (Plant)
ucom_00014787-RA	CDKA1	*Pycnococcus provasolii* (Green microalga)
ucom_00000366-RA	CDKA1	*Vanilla planifolia* (Plant)
ucom_00001449-RA	CDKA1	*Paramecium pentaurelia* (Ciliophora)
ucom_00003817-RA	CDKB1	*Phoenix dactylifera* (Plant)
ucom_00003822-RA	CDKB1	*Phoenix dactylifera* (Plant)
ucom_00003818-RA	CDKB1	*Gossypium tomentosum* (Plant)
ucom_00014855-RA	CDKC2	*Amborella trichopoda* (Plant)
ucom_00003451-RA	CDKC2	*Chloropicon primus* (Green microalgae)
ucom_00012285-RA	CDKC2	*Scyliorhinus canicula* (Animal)
ucom_00000668-RA	CDKD1	*Pimephales promelas* (Animal)
ucom_00000803-RA	CDKD3	*Camelina sativa* (Plant)
ucom_00000804-RA	CDKD3	*Brassica oleracea* (Plant)
ucom_00001516-RA	CDKE1	*Gossypium mustelinum* (Plant)
ucom_00003327-RA	CDKF4	*Mikania micrantha* (Plant)
ucom_00012284-RA	CDKG2	*Ananas comosus* (Plant)
ucom_00019234-RA	CDKG2	*Ananas comosus* (Plant)
ucom_00002855-RA	CDKG2	*Megalops cyprinoides* (Animal)
ucom_00005799-RA	CDKG2	*Gossypium mustelinum* (Plant)
ucom_00015736-RA	CDK1	*Alca torda* (Animal)
ucom_00013138-RA	CDK2	*Chiloscyllium plagiosum* (Animal)
ucom_00007067-RA	CDK2	*Penaeus vannamei* (Animal)
ucom_00006062-RA	CDK7	*Fistulifera solaris* (Diatom)
ucom_00012364-RA	CDK12	*Actinomortierella ambigua* (Fungus)
ucom_00012367-RA	CDK12	*Actinomortierella ambigua* (Fungus)

Table S6. Protein-coding genes involved in cell wall synthesis in *U. compressa*

Gene	Annotation	Best blast hit
ucom_00010296-RA	Cellulose synthase	*Carex littledalei* (Plant)
ucom_00003660-RA	Cellulose synthase	*Coccomyxa sp.* (Green microalga)
ucom_00007288-RA	Cellulose synthase	*Nostoc sp*. (Cyanobacteria)
ucom_00005233-RA	Cellulose synthase	*Demequina globuliformis* (Bacteria)
ucom_00011264-RA	Cellulose synthase	*Leuconostoc sp.* (Bacteria)
ucom_00001428-RA	Cellulose synthase	*Burkholderia ubonensis* (Bacteria)
ucom_00016578-RA	Cellulose synthase	*Bradyrhizobium sp.* (Bacteria)
ucom_00018845-RA	O-arabinosyltransferase	*Gossypium barbadense* (Plant)
ucom_00001778-RA	O-arabinosyltransferase	*Arabidopsis thaliana* (Plant)
ucom_00005155-RA	O-arabinosyltransferase	*Lotus japonicus* (Plant)
ucom_00019066-RA	O-arabinosyltransferase	*Hevea brasiliensis* (Plant)
ucom_00000679-RA	O-arabinosyltransferase	*Hevea brasiliensis* (Plant)
ucom_00000681-RA	O-arabinosyltransferase	*Hevea brasiliensis* (Plant)
ucom_00012754-RA	O-arabinosyltransferase	*Micractinium sp. (*Green microalga)
ucom_00013569-RA	O-arabinosyltransferase	*Chlamydomonas sp.* (Green microalga)
ucom_00012224-RA	Mannosyltransferase	*Arabidopsis thaliana* (Plant)
ucom_00006352-RA	Mannosyltransferase	*Dictyobacter formicarum* (Bacteria)
ucom_00003345-RA	UDP-glucosamine transferase	*Glycine max* (Plant)
ucom_00010669-RA	UDP-rhamnose transporter	*Oryza meyeriana* (Plant)
ucom_00004334-RA	UDP-rhamnose transporter	*Carex littledalei* (Plant)
ucom_00011580-RA	UDP-uronic acid transporter	*Brassica napus* (Plant)
ucom_00010744-RA	UDP-uronic acid transporter	*Brassica napus* (Plant)
ucom_00003648-RA	Pectin lyase	*Micractinium sp.* (Green microalga)
ucom_00009863-RA	Collagen alpha-2	*Culex quinquefasciatus* (Animal)
ucom_00013278-RA	Collagen alpha-2	*Eumeta japonica* (Animal)
ucom_00008150-RA	Collagen alpha-3	*Cervus canadensis* (Animal)
ucom_00015512-RA	Collagen alpha-5	*Bos taurus* (Animal)
ucom_00007898-RA	Extensin	*Drosophila erecta* (Animal)
ucom_00017011-RA	Extensin	*Octodon degus* (Animal)
ucom_00016833-RA	Extensin	*Cyprinodon tularosa* (Animal)
ucom_00014857-RA	Expansin	*Haliotis rubra* (Animal)
ucom_00004669-RA	Elastin isoform X10	*Drosophila subobscura* (Animal)
ucom_00000187-RA	Elastin isoform X10	*Drosophila subobscura* (Animal)
ucom_00001742-RA	Fibronectin	*Nostoc sp.* (Cyanobacteria)
ucom_00006108-RA	Fibronectin	*Chlorella variabilis* (Green microalga)
ucom_00010344-RA	Fibronectin	*Achlya hypogyna* (Oomycete)
ucom_00010351-RA	Fibronectin	*Rodococcus spongiicola* (Bacteria)

Table S7. Protein-coding genes of calcium transport in *U. compressa*

**Gene**	**Annotation**	**Best blast hit**
ucom_00009416-RA	VDCC	*Chlamydomonas sp.* (Green microalga)
ucom_00009418-RA	VDCC	*Chlamydomonas sp.* (Green microalga)
ucom_00004154-RA	VDCC	*Chlamydomonas sp.* (Green microalga)
ucom_00016786-RA	Ryanodine-dependent channel	*Albugo laibachii* (Fungus)
ucom_00018869-RA	Ryanodine-dependent channel	*Astyanax fasciatus* (Animal)
ucom_00012480-RA	Ryanodine-dependent channel	*Albugo laibachii* (Fungus)
ucom_00002757-RA	IP3-dependent channel	*Zea mays* (Plant)
ucom_00007431-RA	IP3-dependent channel	*Gossypium barbadense* (Plant)
ucom_00005839-RA	Glutamate receptor	Hevea brasiliensis (Plant)
ucom_00007076-RA	Glutamate receptor	*Cajanus cajan* (Plant)
ucom_00007082-RA	Glutamate receptor	*Senna tora* (Plant)
ucom_00007074-RA	Glutamate receptor	*Chlorella desiccata* (Green microalga)
ucom_00000458-RA	Glutamate receptor	*Chloropicon primus* (Green microalga)
ucom_00002349-RA	Glutamate receptor	*Micractinium sp.* (Green microalga)
ucom_00007079-RA	Glutamate receptor	*Chlorella desiccata* (Green microalga)
ucom_00019116-RA	Glutamate receptor	*Micractinium sp.* (Green microalga)
ucom_00005273-RA	Glutamate receptor	*Melanotaenia boesemani* (Fish)
ucom_00007085-RA	Glutamate receptor	*Penaeus japonicus* (Animal)
ucom_00009503-RA	Glutamate receptor	*Epinephelus lanceolatus* (Fish)
